# Music Composition from the Brain Signal: Representing the Mental State by Music

**DOI:** 10.1155/2010/267671

**Published:** 2010-03-11

**Authors:** Dan Wu, Chaoyi Li, Yu Yin, Changzheng Zhou, Dezhong Yao

**Affiliations:** ^1^Key Laboratory for NeuroInformation of Ministry of Education, School of Life Science and Technology, University of Electronic Science and Technology of China, Chengdu 610054, China; ^2^Shanghai Institutes for Biological Sciences, Chinese Academy of Sciences, Shanghai 200031, China; ^3^Department of Students' Affairs, Arts Education Centre, University of Electronic Science and Technology of China, Chengdu 610054, China

## Abstract

This paper proposes a method to translate human EEG into music, so as to represent mental state by music. The arousal levels of the brain mental state and music emotion are implicitly used as the bridge between the mind world and the music. The arousal level of the brain is based on the EEG features extracted mainly by wavelet analysis, and the music arousal level is related to the musical parameters such as pitch, tempo, rhythm, and tonality. While composing, some music principles (harmonics and structure) were taken into consideration. With EEGs during various sleep stages as an example, the music generated from them had different patterns of pitch, rhythm, and tonality. 35 volunteers listened to the music pieces, and significant difference in music arousal levels was found. It implied that different mental states may be identified by the corresponding music, and so the music from EEG may be a potential tool for EEG monitoring, biofeedback therapy, and so forth.

## 1. Introduction

Music is a universal human trait throughout the human history and across all cultures, and it is also a powerful tool for emotion and mood modulation [1]. Music is not only a kind of entertainment, but another kind of language; thus music composition may be conceived as a specific representation of human mind. 

Along with the widespread of computer application, some researchers attempted to “teach” the computer to compose music, where a variety of mathematic algorithms [2] and fundamental music rules [3] were explored. In general, for such a computer composition, subjective algorithm design and artificial selection of music rules are crucial and difficult. To learn from the nature and ourselves, various signals from human body, such as the DNA [4], proteins [5], electromyograms (EMGs) [6], and brainwaves [7], have been utilized in computer composition in 1990s. 

The brainwaves, the electroencephalograms (EEGs), are the visual plotting of the brain neural electric activities projected to the scalp surface. The earliest attempt to hear brainwaves as music was made in 1934 [8]. In most of these early works, however, only the amplitude of the alpha waves or other simple and direct characters of EEG signals was utilized as the driving sources of the musical sound. In the 1990s, various new music generating rules were created from digital filtering or coherent analysis of EEG [9]. In general, these techniques may be classified into two categories. The first one is sonification, which aims at monitoring the brainwaves in an auditory way and includes various methods, such as the direct parameter mapping [10], the one based on the interictal epileptic discharges as triggers for the beginning of music tones [11], and rules worked on the scale-free phenomena which exists at both EEG and music [12]. The second one is the brainwave music which has involved musical theories in composition. The typical work is the application of brainwave music in Brain Computer Interface (BCI) [7]. 

In this work, we proposed a method to translate the mental signal, the EEG, into music. The goal is to represent the mental state by music. The arousal levels corresponding to both the brain mental states and music emotion are implicitly used as the bridge between the mind world and music melody. EEGs during various sleep stages were tested as the example.

## 2. Material and Methods

### 2.1. Sleep EEG Data

To show the performance of the proposed mapping rules, we apply this method to the real EEG data recorded during the different sleep stages. The sleep stages were recognized by two of the authors according to the rules of Rechtschaffen and Kales (R&K). The data of rapid-eye movement sleep (REM) and nonrapid eye movement were utilized. For the nonrapid eye movement sleep data, we chose segments from both stage 2 (named NREM henceforth) and stages 3 or 4 (the slow-wave sleep (SWS)). The subject was a 25-year-old male, physically and mentally healthy, right-handed. The study was approved by the local Review Board for Human Participant Research. The subject signed an informed consent form for the experiment. The signals were recorded by a 32 channel NeuroScan system with a sampling rate of 250 Hz and were band-pass filtered from 0.5 Hz to 40 Hz. The data is referenced to infinity [13]. The data for music generation is acquired from the second night of the subject sleeping with the braincap. The following analysis was performed on the data at electrode Cz, which is at the central of the head and is a channel less affected by the body movement.

### 2.2. EEG, Music, and Arousal

#### 2.2.1. Sleep EEG and Mental Arousal Level

It is believed that the arousal level in different sleep stages is associated with the brain activities; it means that a sleep stage, REM, NREM, or SWS, is a phenomenological representation of the underlying neural activities which are the electrophysiological reflection of a specific mental arousal state. For example, REM is considered to be deeply related to dreams, which involves learning and memory [14]; thus it is considered being more alert than SWS; that is, it has a high arousal level than SWS. 

The time-frequency EEG features are different among REM, NREM, and SWS. The REM stage shows small amplitude activities, similar to light drowsiness, and its alpha band (8–13 Hz) activity is slightly slower than in wakefulness. The brainwaves of SWS are of quite preponderant delta waves (1–4 Hz) and theta rhythm (4–7 Hz), thus typically of low frequency and high amplitude. And the wave amplitude and frequency of NREM are between REM and SWS. As the sleep stages can be identified by the features of EEG, which may be utilized as the physiological indexes of different mental states for music composition of various arousal levels.

#### 2.2.2. Music and Emotion Arousal

As a representation of the internal emotion of a composer, music with some features can be adopted to evoke emotion and mood state. Some studies indicated that music emotion is able to be communicated with various acoustic cues, including tempo, sound level, spectrum, and articulation [15]. And musical structures or patterns usually have their inherent emotional expressions [16]. To evaluate music emotion, a popular one is the Thayer's model, which describes emotion in two dimensions, the valence and the arousal. The valence indicates whether the music is pleasant or unpleasant, while the arousal represents the activity of the music, the activeness or passiveness of the emotion [17]. The two-dimension structure gives us important cues for computational modeling.

Therefore, the musical structure and some features such as pitch, tonality, rhythm, and volume played important roles in the emotion expression. For example, a fast tempo (dense rhythm cadence) usually represents a high arousal level, while a slow tempo (sparse rhythm cadence) indicates the low arousal emotion [18].

### 2.3. Music Generation from EEG

**Figure 1:**
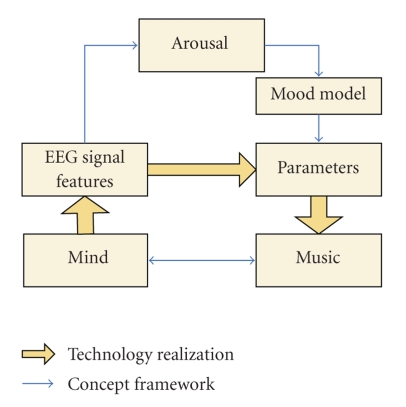
Overview of the brainwave music generation.

For music generation, the overview of the method is shown in Figure 1, where the blue arrow indicates the conceptual framework and the yellow arrow shows the actual realization in this work. Using arousal as a bridge, EEG features were extracted as a reflection of the mind state, and it was mapped to the parameters of music which had similar arousal level according to the two-dimension mood model.

**Figure 2:**
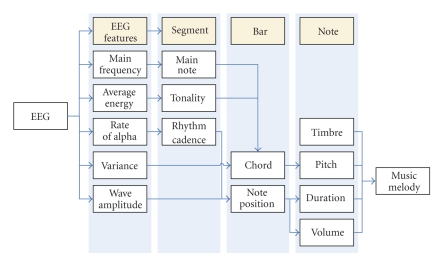
Mapping rules from EEG to music.

The music generation consists of five steps, details shown in Figure 2. First, extract the EEG signal features; second, define the music segments (parameters: *main note, tonality, *and* rhythm cadence*) based on the corresponding EEG features; third, generate music bars (parameters: *chord* and *note position*) from the EEG features and music segment parameters; fourth, fix the values of notes (*timbre, pitch, duration,* and *volume*) according to the bar parameters; the last, construct the music melody by a software (Max/MSP) and a MIDI file is made.

#### 2.3.1. EEG Features and Music Segment

For different mental states, EEGs are of distinct features in frequency and amplitude, that is, different time-frequency (T-F) patterns. The *main frequency*, *rate of alpha,* and *variance* are obtained from the complex morlet wavelet coefficients, while the *wave amplitude* and *average energy* are estimated directly from the EEG signal. 

The music sequence has the same time length as the real EEG. The segmentation is based on the following inequation (1), so that when it exists, a new segment is assumed:

(1) $$\frac{\left|x\left(i\right)-\overset{̅}{x}\right|}{\overset{̅}{x}}>1,$$

where  *x*(*i*)  denotes the value of the EEG signal at the current point *i,* and $\overset{̅}{x}$ is the average of the data *x*(*i*) from the last segment ending to the current time.

In a segment, the three parameters, *main note, tonality,* and *rhythm*, are kept the same. As shown in Figure 2, the *main note*, the most important note in a music melody, is based on the EEG *main frequency*. When the EEG *main frequency* is high, the *main note* is high, and vice versa. 

According to music esthetic theory about *tonality*, a major music usually is utilized for a positive melody, while a minor is identified to be soft and mellow [18]. In this work, we defined an empirical threshold that when the average energy is lower than the threshold, we take the Major; else the Minor. Therefore, a deep sleep stage, SWS, would be represented by a minor key, and the REM stage would be matched with the major. The key transfer would make the music pieces have a rich variety, and the stage change can be identified by the music modulation.

The *rhythm cadence* is related to the *rate of alpha*. When it is high, the rhythm cadence is dense, which means that the number of notes in a fixed length is large. The result is that a fast tempo corresponds to a high arousal level. When the rate is low, the condition is adverse.

#### 2.3.2. Music Generation: Bar

In a music segment, the substructure is the bar, where the chord and note position are finally determined. As variance of the wavelet coefficients can represent the change of the frequency combination in the time-frequency domain, we use it to determine the chord. Since the chord is complex, here we simply assume that the stability of the chord and the change of EEG spectrum are consistent.

In this work, we take 4 beats in a bar and 4 positions in a beat. The parameter “*note position*” indicates a note on or off at a position. The *rhythm cadence* determines the number of notes on, and then the EEG amplitude over an empirical threshold for each bar determines the specific position for a note “on.”

#### 2.3.3. Music Generation: Note

Music melody is the combination of notes, and for each note, there are four essential features: timbre, pitch, duration, and volume. 

The *timbre* of the note is assumed to be piano in this work. And in general, we may have different timbres for different segments if necessary.

The *pitch* of a note is related to the chord. In our method, each bar has a chord, and the notes in a bar are selected from two families: the first family consists of the note pitches of the harmonic chord (chord tone) and the second includes the other note pitches of the scale (none chord tone). For example, when the chord is major C, the family of chord tone consists of C, E, G, while the none chord tone family includes D, F, A, B. To ensure the tonality of the melody, there are a few rules for pitch family choice; for example the chord notes are usually placed at the downbeat (the first beat of the bar); the pitch interval is limited to 7 semitones.

The *duration* of a note is represented by the note position. A note starts at the position of a note on and lasts until the next note-on position. However, the lasting must be in the same bar so that if the next note-on position is in the next bar, the current note's duration will stop at the current bar end.

The *volume* of a note is indicated by the note position of the beat. A downbeat has a large volume, while an upbeat has a small volume.

#### 2.3.4. Music Emotion Evaluation Test

In order to ascertain if the music of different sleep states can be identified, and to see the emotion dimensions when people listen to them, 35 healthy students (20 males, 15 females), ranging in age from 21 to 28 years (mean 22.49, SD 1.65), were asked to participate in this test. None of the volunteers reported any neurological disorders, psychiatric diseases, or were on medication. All had normal hearing. 94.3% of them had never received special musical education, and 5.7% of them (two subjects) had special musical training less than 2 years. 

**Figure 3:**
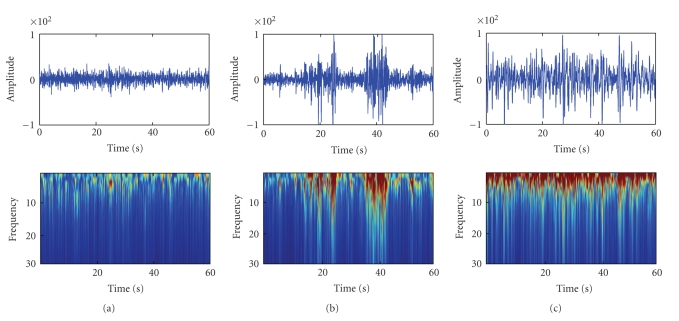
Sleep EEG and Wavelet analysis. (a) REM; (b) NREM; (c) SWS.

Since the waveforms of REM and SWS are more typical than NREM (see Figure 3), we designed a test with 10 music pieces consisting of 5 from REM and 5 from SWS with the proposed mapping rule. Each music piece lasted 60 seconds and they were randomly played to the volunteers. The volunteers were asked to focus on the emotions of the music pieces. After listening to each piece, they were required to mark a table for the arousal levels which had a 9-point scale from 1 to 9 (with “1 = very passive” and “9 = very excited” on the arousal scale).

## 3. Results

### 3.1. Music of Sleep EEG

Figure 3 shows the wavelet analysis results of REM, NREM, and SWS. Apparently, the REM and SWS data may be assumed to be one segment, while the NREM data should be segmented into five segments for its very clear variety of features in frequency and amplitude related to the spindle waves. For the data in Figure 3, we found that segment 1 of NREM was quite similar to REM, segment 2 is similar to SWS, and the reason is that the wave amplitude and frequency of NREM are between REM and SWS as noted above.

**Figure 4:**
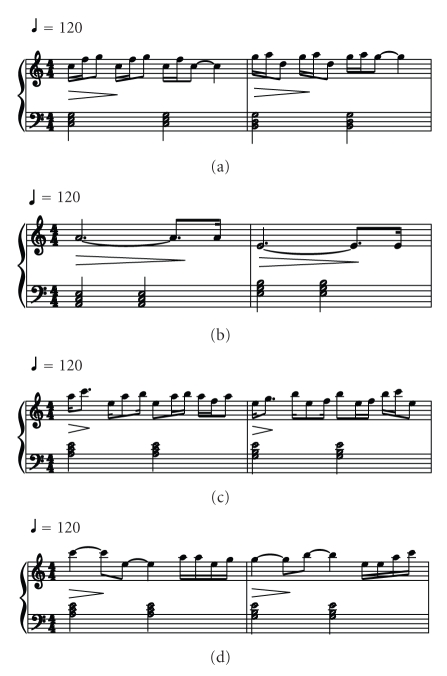
Music scores for REM (a), SWS (b), NREM segment 1 (c), and NREM segment 2 (d).

 The music pieces of different sleep stages are of different features in music structure. Table 1 shows the music parameters of REM and SWS EEG. The REM music has high-pitch notes and dense rhythm; thus it indicates a high arousal state. The SWS music has notes of low pitch, and the rhythm is sparse; thus it denotes a low arousal state. Figure 4 shows the examples of the music scores of the sleep EEG.

### 3.2. Music Emotion Evaluation

**Figure 5:**
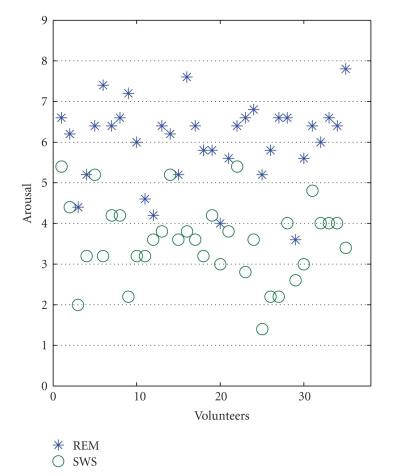
The distribution of the emotion arousal levels of the REM and SWS music.

In the music emotion evaluation test, the arousal level of REM and SWS is 6.02 ± 0.99 and 3.59 ± 0.97, respectively. And the differences between them are significant (T(34) = 12.571, *P* < .01). Figure 5 showed the points from all the volunteers in the emotion space with blue stars and green circles for the REM and SWS music pieces, respectively. It is quite clear that the music of REM has high arousal level than SWS, which means that the music of REM is more active. Figure 5 demonstrates that our method can translate the different arousal mental state to the corresponding music arousal level. The arousal level of REM music is higher than SWS music for all the listeners, although their absolute arousal level points are different.

## 4. Discussions and Conclusion

There is growing interest in the relation between the brain and music. The approach to translate the EEG data into music is an attempt to represent the mind world with music. Although arousal has been a common topic in both brain mental state and music emotion studies, it is a new attempt to use arousal as the bridge between the brain mental state and the music. The above results show that the approach is advisable and effective and that the music pieces of different sleep stages are of distinct music features corresponding to the different levels of arousal. The active state is represented by music pieces with high arousal level, while music for the calm state has a low arousal level. 

In this EEG music generation, some basic music theories have been considered. As EEG is a physiologic signal, if we translate it into music directly, the music may be stochastic; if the music rules are too strictly followed, some detailed meaningful EEG information may be ignored. To keep a proper balance between the science (direct translation) and art (composition), only some important principles of music were involved in the program, and the features of the EEG were chosen carefully to maintain the most meaningful physiologic information. If some random parameters are utilized to replace these features, the music would show no specific patterns. In general, the choice of the feature extraction method would influence the meaning of the derived music, and any principle followed by both the brain activity and music would be an appropriate bridge between the brainwave and music. 

In this pilot experiment, the method was evaluated on the sleep EEG data from one subject. Though individual EEG data is different from one subject to another, the basic features of sleep EEG with different mental states are quite steady, such as the characteristic waves of different sleep stages. That means, for the same sleep stage, that the music of different subjects would be different in details, but the main patterns would be similar. 

To improve this work, other EEG signal processing methods can be adopted, such as complexity analysis, independent component analysis, and fractal analysis (power law [12]). In our current method, we just consider the arousal level of the brain and music while the other emotion dimensions, such as valence, can also be involved in the further music generation studies. Moreover, the program needs to be tested on more data to improve itself to adapt to various cases. 

This method might be used potentially in an assistant sleep monitor in clinical applications because the music of different sleep stages can be identified easily and more comfortably. However, it needs further experimental studies before any practical application. Also, it can work as a musical analytical method for the ample states of EEG. Furthermore, this method can be utilized as a unique automatic music generation system, which enables those people who have no composition skills to make music through using their brainwaves. Therefore, it can be utilized as a bio-feedback tool in disease therapy and fatigue recovery.

## Figures and Tables

**Table 1:** Music parameters of REM and SWS.

Music parameters	Main note	Tonality	Rhythm cadence	Pitch	Duration	Volume
REM	High	Major	Dense	High	Short	Large
SWS	Low	Minor	Sparse	Low	Long	Small
